# Surgical Release of a Calcified Conjoint Tendon Following Reverse Total Shoulder Arthroplasty: A Case Report

**DOI:** 10.1155/cro/3070924

**Published:** 2026-03-23

**Authors:** Andrew Gaetano, Michael Scheidt, Jason Howard, Andrew Chen, Nickolas Garbis, Dane Salazar

**Affiliations:** ^1^ Stritch School of Medicine, Loyola University Medical Center, Maywood, Illinois, USA, loyolamedicine.org; ^2^ Department of Orthopaedic Surgery and Rehabilitation, Loyola University Health System, Maywood, Illinois, USA, loyolamedicine.org

**Keywords:** anterior shoulder pain, calcific tendinopathy, case report, conjoint tendon, reverse total shoulder arthroplasty, surgical release

## Abstract

A 69‐year‐old female with a history of reverse total shoulder arthroplasty presented with persistent anterior shoulder pain and restricted range of motion. Imaging subsequently demonstrated calcific changes of the conjoint tendon. Targeted corticosteroid injections provided temporary symptom relief; however, recurrence after discontinuation due to steroid‐induced hyperglycemia prompted surgical tendon release. Postoperatively, range of motion improvements were sustained, but anterior shoulder pain recurred and Quick‐DASH scores worsened by the 6‐month follow‐up. Although calcific changes were identified within the conjoint tendon, the clinical course suggests that calcification may represent a contributing or secondary phenomenon rather than a definitive primary pain generator. Conjoint tendon release may improve mobility, although pain relief may be unpredictable.

## 1. Introduction

Reverse total shoulder arthroplasty (RTSA) is the most common form of shoulder replacement [1, 2]. Its indications have broadened and include rotator cuff arthropathy, severe glenohumeral osteoarthritis, and complex proximal humerus fractures [3, 4]. Despite favorable outcomes, RTSA is associated with complications including infection, instability, implant loosening, neurologic injury, scapular notching, and fractures of the glenoid, acromion, or scapular spine [2, 5, 6].

Persistent pain following RTSA is a recognized complication and may indicate implant malposition, scapular notching, component loosening, or soft tissue fatigue [7–9]. One potential source of anterior shoulder pain is tensioning of the conjoint tendon, comprising the short head of the biceps brachii and the coracobrachialis, due to lengthening from inferiorization and medialization of the glenohumeral center of rotation in Grammont‐style prostheses [4, 10]. This increased tension may lead to tendonitis and chronic anterior shoulder pain. Open conjoint tendon release has been reported to improve symptoms in select patients [9].

Calcific tendinopathy is characterized by deposition of calcium hydroxyapatite crystals within tendon tissue leading to pain and stiffness, and it is most commonly described in the rotator cuff and long head of the biceps [11, 12]. Its pathogenesis is incompletely understood but may involve mechanical stress, vascular impairment resulting in local hypoxia, fibrocartilaginous metaplasia, and subsequent mineralization [13, 14]. Diagnosis is typically radiographic, with CT occasionally used for further delineation.

Although calcific tendinopathy is well described in other shoulder tendons, involvement of the conjoint tendon has not been previously reported to our knowledge. We present a case of calcific changes within the conjoint tendon identified in a patient with persistent anterior shoulder pain following RTSA. Although a direct causal relationship cannot be definitively established, this case highlights a potential association that warrants further investigation.

## 2. Case Presentation

Given that this was a case report of a single patient, Institutional Review Board approval of this study was waived. The patient provided consent for participation and publication. This case report was prepared in accordance with the CARE (CAse REport) guidelines.

### 2.1. History and Physical Exam

A 69‐year‐old female with diabetes mellitus, hypertension, liver disease, and thyroid cancer presented with persistent right anterior shoulder pain and tightness 15 months after RTSA for rotator cuff tear arthropathy using a Zimmer Trabecular Metal Reverse Shoulder System (Zimmer Biomet, Warsaw, Indiana). At presentation, the patient′s pain was rated 3/10 on the visual analog scale (VAS).

Examination revealed tenderness to palpation over the subcoracoid and coracoid regions. Active range of motion (ROM) was 140° of forward flexion, 25° of adducted external rotation, and internal rotation to the waist. Laboratory evaluation (ESR, CRP, and WBC) was normal, and joint aspiration ruled out periprosthetic infection. Electromyography confirmed intact axillary nerve function.

A fluoroscopy‐guided corticosteroid injection at the conjoint tendon resulted in complete symptom relief and improved ROM, supporting the involvement of this region in symptom generation. However, after two additional injections over the following year, treatment was discontinued due to steroid‐induced hyperglycemia, necessitating cessation of further corticosteroid therapy. Following discontinuation of injections, the patient′s anterior shoulder pain recurred.

Plain radiographs of the right shoulder demonstrated stable implant alignment with subtle subcoracoid calcifications (Figure 1). CT demonstrated discrete hyperattenuating intratendinous foci adjacent to the coracoid, consistent with calcific or ossific changes within the conjoint tendon (Figure 2). Given the patient′s persistent symptoms despite comprehensive clinical work‐up and failure of conservative treatment, surgical management with open release of the conjoint tendon was recommended. A timeline summarizing the patient′s clinical course, imaging studies, and interventions is presented in Table 1.

Figure 1Grashey (a), scapular y (b), and axillary (c) plain radiographs of the right shoulder demonstrating a Grammont style reverse total shoulder prosthesis with stable alignment preconjoint tendon release. There is no apparent subsidence or loosening. Subcoracoid calcifications are apparent, consistent with enthesopathy of the conjoint tendon.

**Figure:**
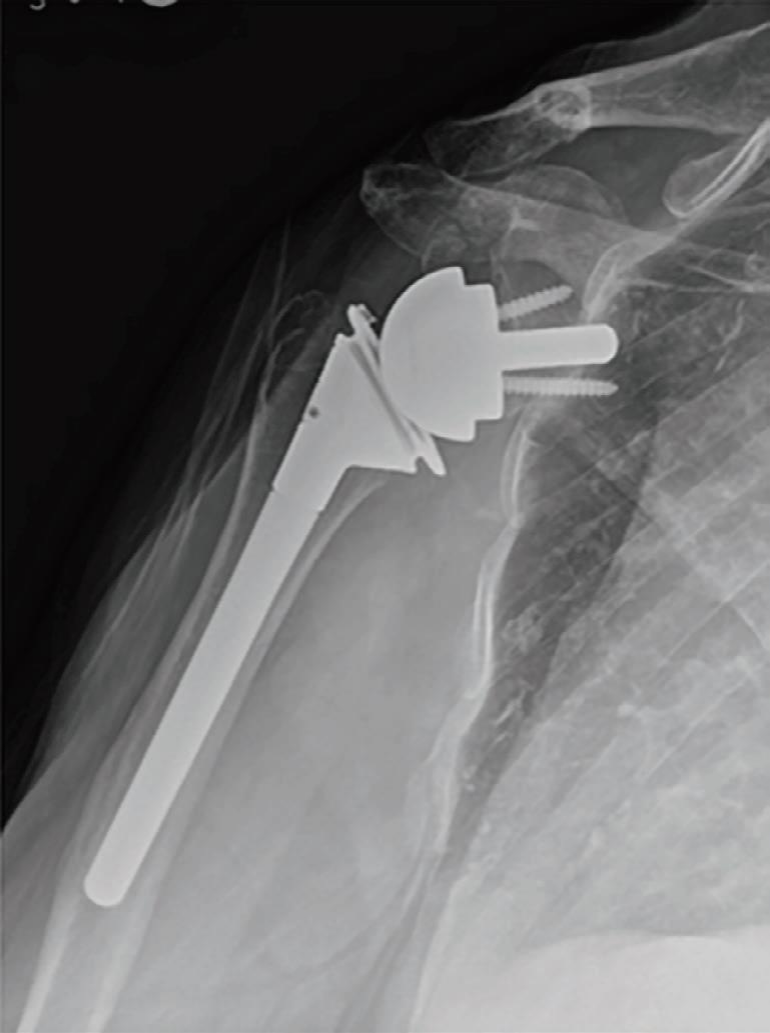
(a)

**Figure:**
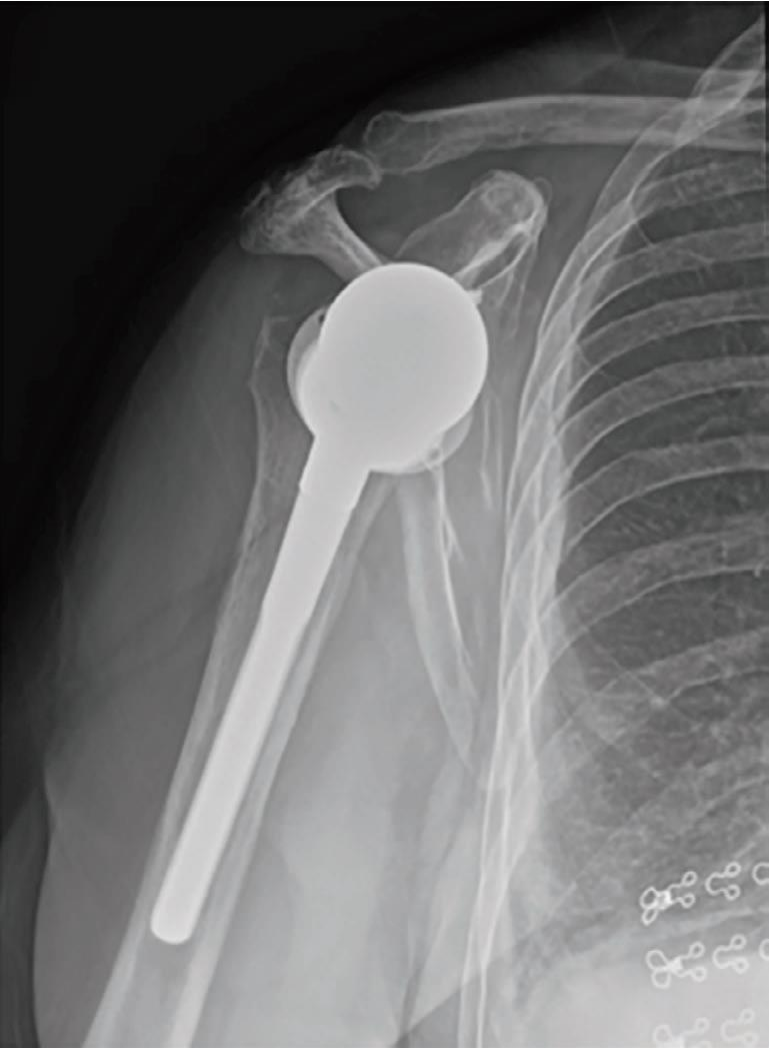
(b)

**Figure:**
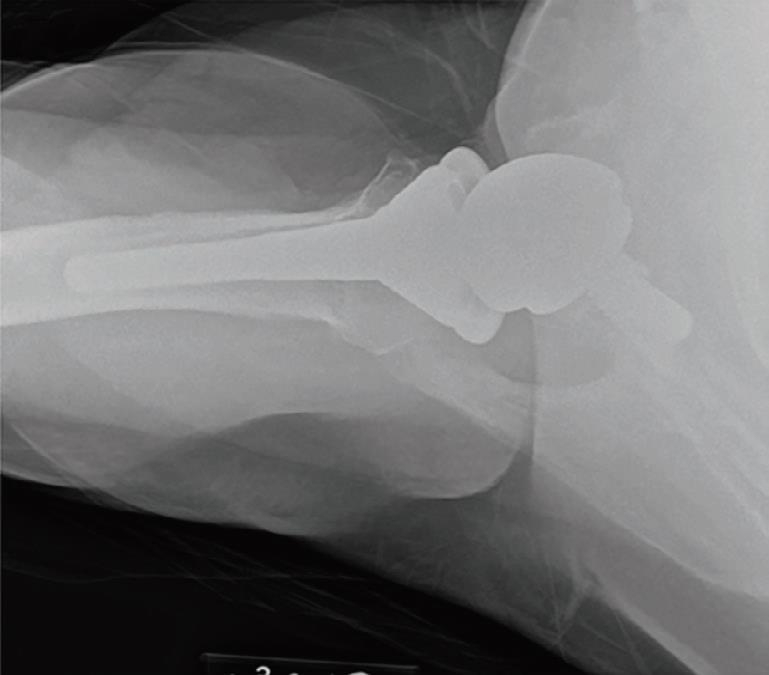
(c)

Figure 2Axial (a) and sagittal (b) computed tomography (CT) images of the right shoulder preconjoint release demonstrating subcentimeter calcific fragments adjacent to the coracoid within the conjoint tendon (red arrow).

**Figure:**
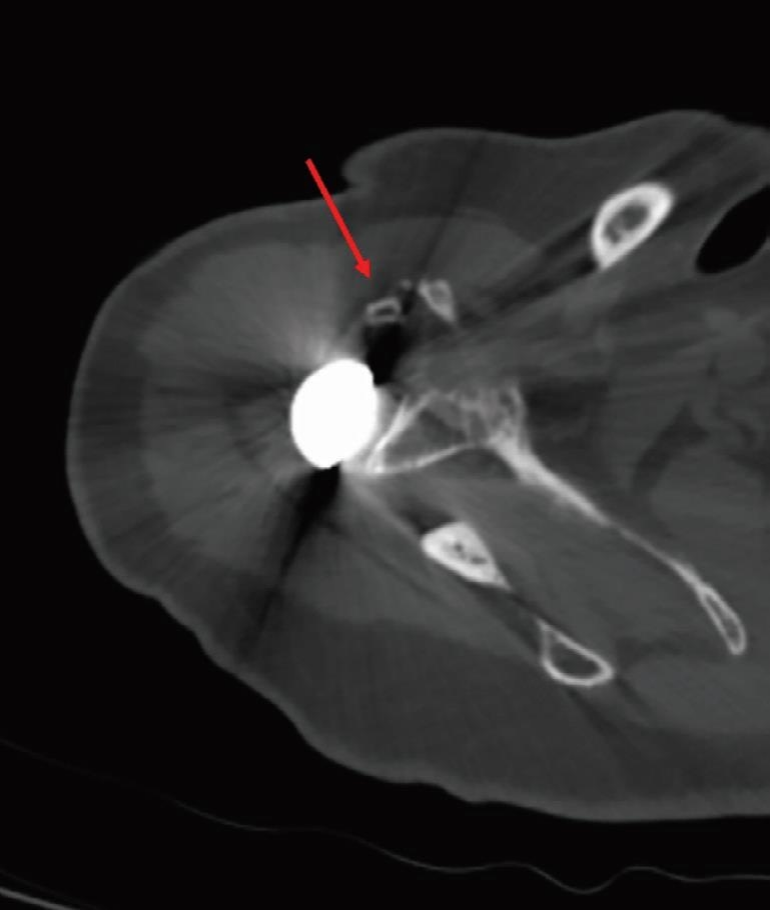
(a)

**Figure:**
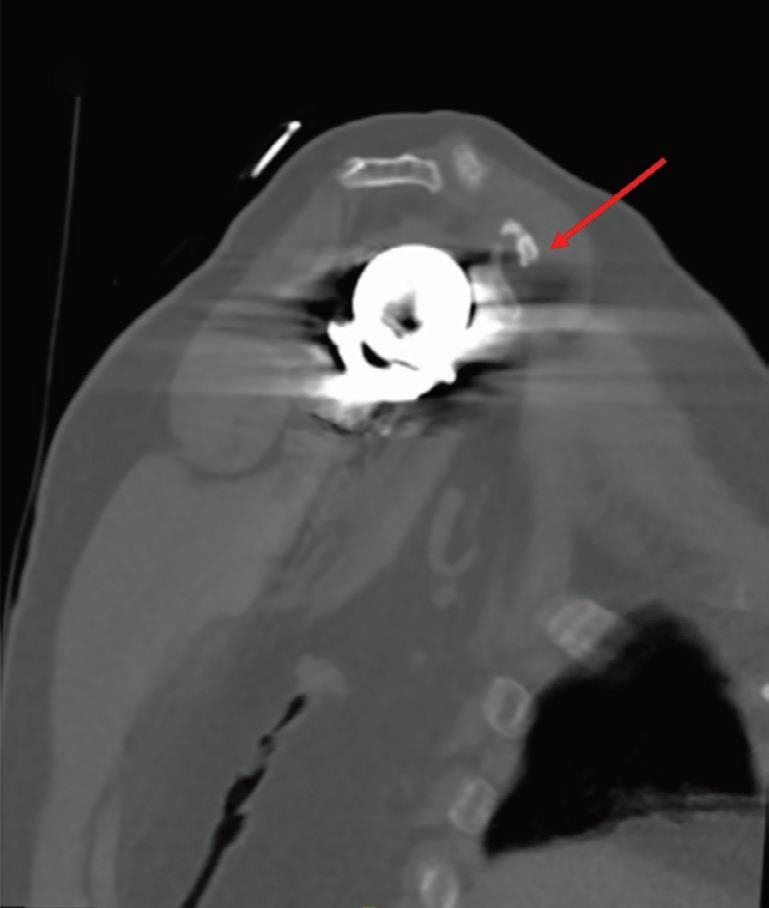
(b)

**Table 1:** Timeline of clinical events following reverse total shoulder arthroplasty.

Time from index RTSA	Key clinical event
15 months	Onset of persistent anterior shoulder pain and tightness; negative laboratory workup, joint aspiration ruled out periprosthetic infection, and electromyography confirmed intact axillary nerve function
15 months	CT scan demonstrating ossific changes within the conjoint tendon adjacent to the coracoid
20 months	Fluoroscopy‐guided corticosteroid injection to the conjoint tendon—marked symptomatic improvement lasting approximately 2 months
23 months	Repeat corticosteroid injection—moderate symptomatic improvement lasting approximately 3–4 months
29 months	Third corticosteroid injection—discontinued due to steroid‐induced hyperglycemia

### 2.2. Surgical Technique

The proximal 4 cm of the patient′s prior deltopectoral incision was utilized, located just inferior and lateral to the tip of the coracoid. A deltopectoral skin incision was made, and full‐thickness skin flaps were developed. The cephalic vein was identified and retracted laterally with the deltoid muscle. The deltoid was swept off the proximal humerus, and the pectoralis muscle was retracted medially. The tip of the coracoid was exposed, and a blunt Homan was placed superiorly to retract the soft tissue. Care was taken to avoid excessive medial dissection in order to protect the musculocutaneous nerve as it courses deep to the conjoint tendon. Retractors were placed directly on bone to minimize soft tissue traction, and attention was given to coracoid osteology to avoid iatrogenic fracture during tendon release. Meticulous hemostasis was achieved prior to closure. At this point, the proximal portion of the conjoint tendon appeared grossly calcified. The tendon was fully released from its origin at the coracoid and retracted distally by 1–2 cm. The involved segment was resected and sent for pathological evaluation, which demonstrated ossified tissue consistent with dystrophic calcification and osseous metaplasia within the conjoint tendon.

### 2.3. Postoperative Protocol

Postoperatively, the patient was immobilized in a sling for comfort only. At the 2‐week follow‐up, she began formal physical therapy focused on active ROM and progressive strengthening without restrictions.

### 2.4. Postoperative Course

At 2 weeks postoperatively, the patient reported a Quick‐DASH (disabilities of the arm, shoulder, and hand) score of 95. She demonstrated 150° of active forward flexion, 40° of adducted external rotation, and internal rotation to the lumbosacral junction. By 4 weeks, her Quick‐DASH score improved to 61. By 12 weeks postoperatively, she showed continued functional improvement with a VAS pain score of 0/10, a Quick‐DASH score of 64, and active forward flexion to 160°. At the 6‐month follow‐up, ROM and strength were maintained; however, she developed unanticipated recurrence of mild anterior shoulder pain and crepitus with movement, without evidence of instability. By 12 months postoperatively, anterior shoulder pain had worsened to 4/10 on the VAS, and her Quick‐DASH score increased to 71. ROM remained stable with 160° of active forward elevation, 40° of external rotation, and internal rotation to the posterior superior iliac spine. Radiographic evaluation showed a well‐positioned implant without evidence of subsidence, breakage, or loosening, and the patient was concentrically reduced without any evidence of scapular notching or adduction impingement (Figure 3). At 18 months postoperatively, the patient remained adherent to formal physical therapy due to persistent anterior shoulder pain, with VAS and Quick‐DASH scores similar to those at the 12‐month follow‐up. Table 2 demonstrates trends in patient‐reported outcome measures and shoulder ROM across follow‐up following conjoint tendon release.

Figure 3Grashey (a), scapular Y (b), and axillary (c) plain radiographs of the right shoulder demonstrating stable alignment 1 year following conjoint tendon release. There is no apparent subsidence or scapular notching.

**Figure:**
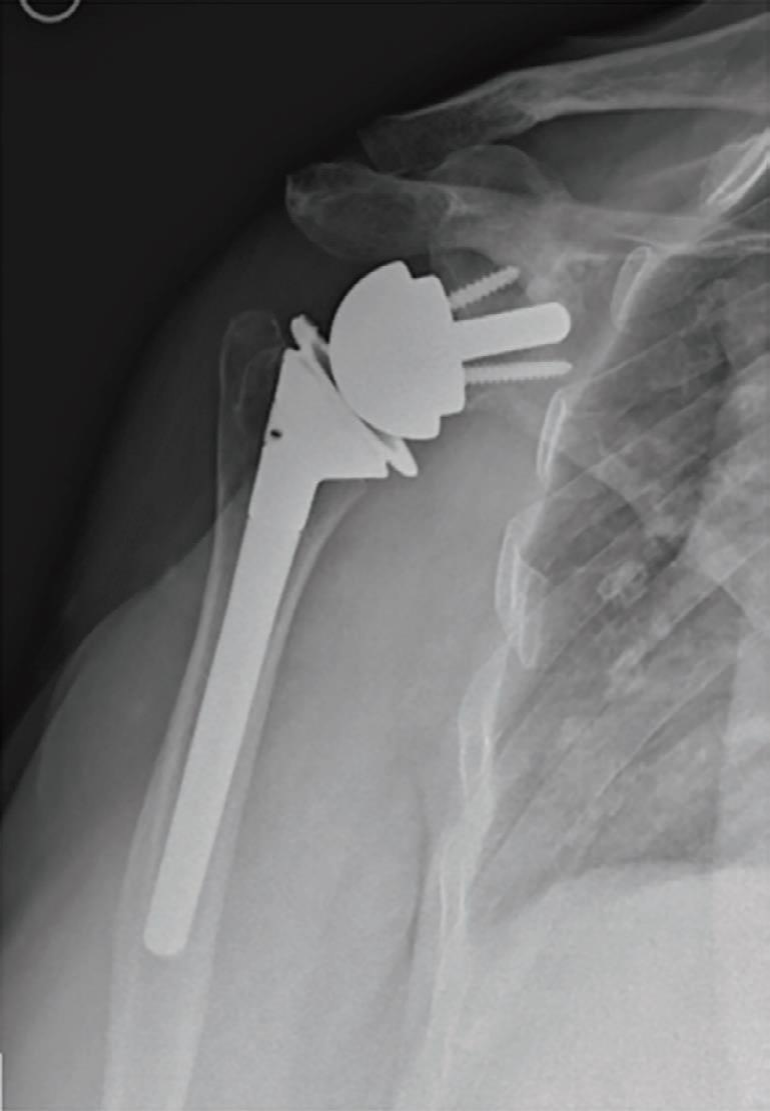
(a)

**Figure:**
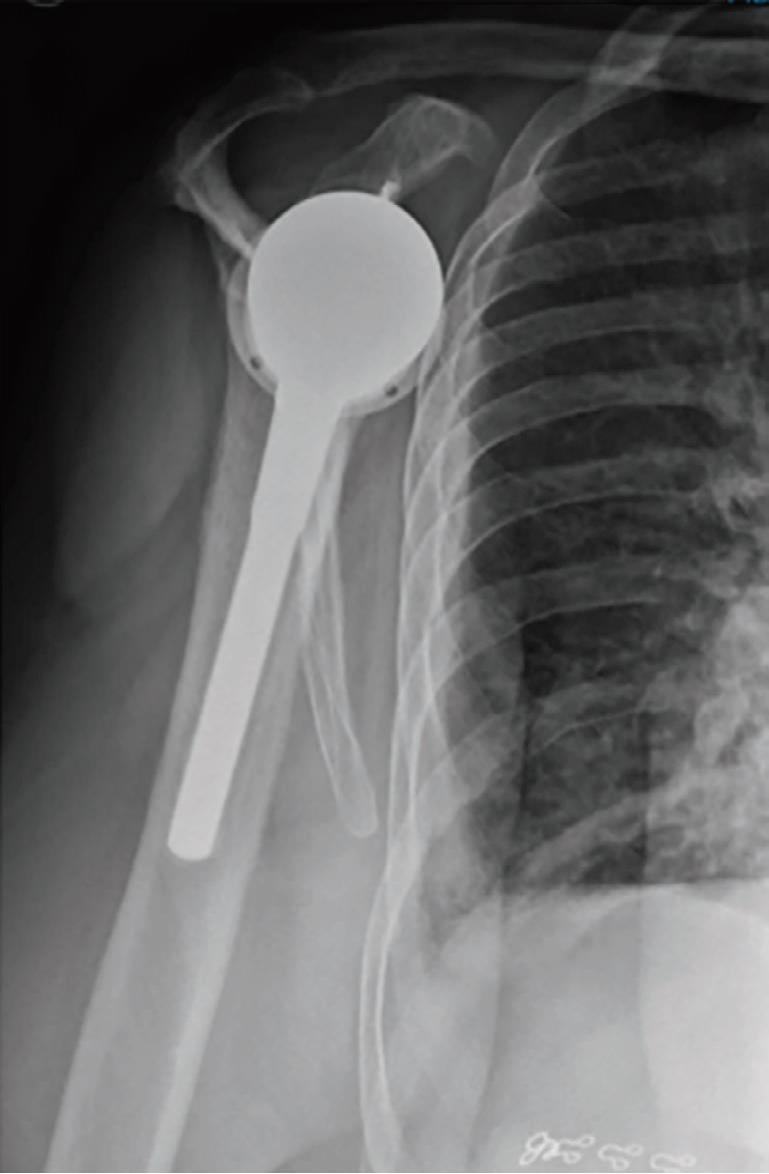
(b)

**Figure:**
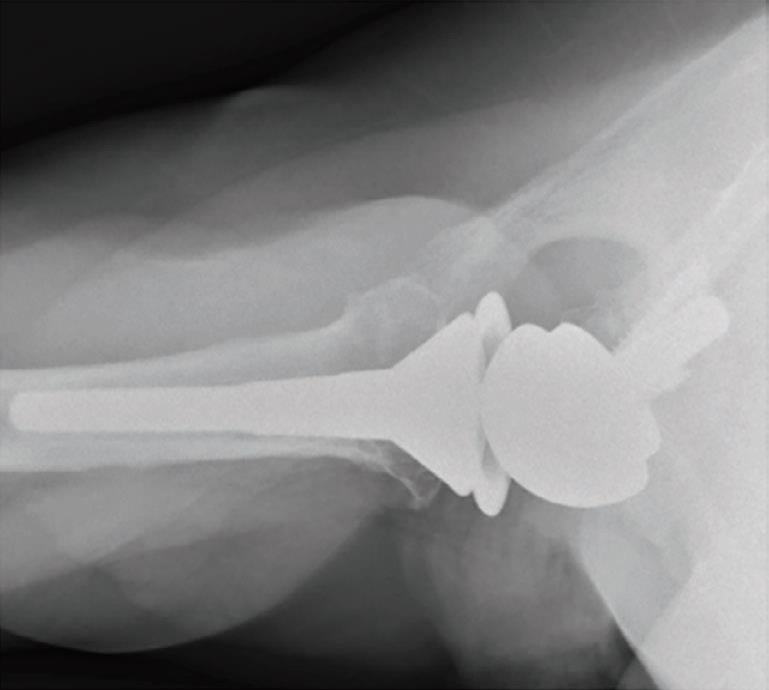
(c)

**Table 2:** Longitudinal changes in patient‐reported outcome measures and shoulder range of motion following open conjoint tendon release.

Time from tendon release	VAS	Quick‐DASH	Range of motion
2 weeks	Not recorded	95%	150° of active forward flexion, 40° of adducted external rotation, and internal rotation to the lumbosacral junction
4 weeks	Not recorded	61%	160° of active forward elevation, 40° of adducted external rotation, and internal rotation to the thoracolumbar junction
12 weeks	0/10	64%	160° of active forward elevation, 40° of adducted external rotation, and internal rotation to the thoracolumbar junction
6 months	1/10	61%	160° of active forward elevation, 40° of adducted external rotation, and internal rotation to the lumbosacral junction
12 months	4/10	71%	160° of active forward elevation, 40° of adducted external rotation, and internal rotation to the posterior superior iliac spine
18 months	4/10	71%	160° of active forward elevation, 45° of adducted external rotation, and internal rotation to the posterior superior iliac spine

## 3. Discussion

RTSA is widely performed with broad indications [3, 4, 15]. Although many postoperative complications are well characterized, persistent anterior shoulder pain remains less clearly understood [2, 5, 16]. Common causes of anterior pain following RTSA include periprosthetic infection, implant malposition or loosening, scapular notching or impingement, instability, acromial or coracoid fracture, neurologic injury, and soft tissue tension or fatigue. In the present case, infection was excluded by normal inflammatory markers and negative joint aspiration, implant‐related complications were ruled out by stable radiographic alignment without evidence of loosening or notching, and neurologic injury was excluded by intact axillary nerve function on electromyography. These findings narrowed the differential diagnosis toward a localized soft tissue etiology in the subcoracoid region.

Tashjian et al. investigated persistent anterior shoulder pain after RTSA and reported outcomes following isolated release of the tensioned conjoint tendon [9]. This study demonstrated significant pain reduction in 9 of 11 patients and improvements in American Shoulder and Elbow Surgeons (ASES) scores in 5 of 11 patients. These findings support the use of conjoint tendon release to manage anterior shoulder pain post‐RTSA.

In this report, calcific changes within the conjoint tendon were identified in a patient with persistent anterior shoulder pain following RTSA. Although intraoperative findings and pathologic evaluation confirmed tendon calcification, the postoperative clinical course introduces uncertainty regarding the degree to which calcification itself served as the primary pain generator. Although short‐term pain relief was achieved and ROM improvements were sustained, anterior shoulder pain recurred and patient‐reported functional scores worsened during mid‐ to long‐term follow‐up. This discordance suggests that calcification may have represented a contributing or secondary phenomenon rather than the sole causative factor. It also underscores that conjoint tendon release may improve mechanical restriction while providing less predictable pain relief, highlighting the importance of evaluating both objective and patient‐reported outcomes when defining surgical success.

The biomechanics of Grammont‐style RTSA may provide a plausible explanation for calcification in this setting. Inferiorization of the center of rotation increases tensile forces across the conjoint tendon [4, 10]. Chronic tensile overload and repetitive microtrauma may promote fibrocartilaginous metaplasia and subsequent calcium hydroxyapatite deposition. Additionally, this patient′s diabetes mellitus may have predisposed her to tendon degeneration and aberrant mineralization, as metabolic dysregulation has been associated with impaired tendon healing and increased risk of calcific tendinopathy [13, 14].

## 4. Conclusion

Calcific changes of the conjoint tendon may be observed in patients with persistent anterior shoulder pain following RTSA and could warrant consideration during evaluation. However, based on this single case, calcification cannot be definitively established as the primary pain generator. Surgical release may improve ROM, although pain relief may be variable. Further investigation is needed to clarify the clinical significance of conjoint tendon calcification in this population.

## Funding

No funding was received for this manuscript.

## Disclosure

The authors, their immediate families, and any research foundation with which they are affiliated have not received any financial payments or other benefits from any commercial entity related to the subject of this article.

## Ethics Statement

Institutional review board approval was not required for this case report.

## Consent

Written informed consent for publication of clinical details and images was obtained; the signed form is held by the institution and is available on request.

## Conflicts of Interest

The authors declare no conflicts of interest.

## Data Availability

Data sharing is not applicable to this article as no datasets were generated or analyzed during the current study.
